# A Review of Synthesis and Applications of Al_2_O_3_ for Organic Dye Degradation/Adsorption

**DOI:** 10.3390/molecules28237922

**Published:** 2023-12-04

**Authors:** Sundarakannan Rajendran, Geetha Palani, Vigneshwaran Shanmugam, Herri Trilaksanna, Karthik Kannan, Marek Nykiel, Kinga Korniejenko, Uthayakumar Marimuthu

**Affiliations:** 1 Institute of Agricultural Engineering, Saveetha School of Engineering, Saveetha Institute of Medical and Technical Sciences, Chennai 602105, India; sundarakannan.r@gmail.com (S.R.); kesangee@gmail.com (G.P.); 2 Department of Mechanical Engineering, Saveetha School of Engineering, Saveetha Institute of Medical and Technical Sciences, Chennai 602105, India; s.vigneshwaren@gmail.com; 3 Department of Physics, Faculty of Advanced Technology and Multidiscipline, Universitas Airlangga, Surabaya 60115, Indonesia; herri-t@fst.unair.ac.id; 4 Chemical Sciences Department and the Radical Research Center, Ariel University, Ariel 40700, Israel; karthikkannanphotoche@gmail.com; 5 Faculty of Material Engineering and Physics, Cracow University of Technology, Jana Pawła II 37, 31-155 Cracow, Poland; marek.nykiel@pk.edu.pl; 6 Faculty of Mechanical Engineering, Kalasalingam Academy of Research and Education, Krishnankoil 626126, India

**Keywords:** dye-sensitized solar cells, environmental applications, sustainable development, Al_2_O_3_, dye degradation, dye adsorption

## Abstract

This comprehensive review investigates the potential of aluminum oxide (Al_2_O_3_) as a highly effective adsorbent for organic dye degradation. Al_2_O_3_ emerges as a promising solution to address environmental challenges associated with dye discharge due to its solid ceramic composition, robust mechanical properties, expansive surface area, and exceptional resistance to environmental degradation. The paper meticulously examines recent advancements in Al_2_O_3_-based materials, emphasizing their efficacy in both organic dye degradation and adsorption. Offering a nuanced understanding of Al_2_O_3_’s pivotal role in environmental remediation, this review provides a valuable synthesis of the latest research developments in the field of dye degradation. It serves as an insightful resource, emphasizing the significant potential of aluminum oxide in mitigating the pressing environmental concerns linked to organic dye discharge. The application of Al_2_O_3_-based catalysts in the photocatalytic treatment of multi-component organic dyes necessitates further exploration, particularly in addressing real-world wastewater complexities.

## 1. Introduction

Solar panels, including dye-sensitized solar cells (DSSCs), are considered a sustainable source of renewable energy and a product that brings many benefits to the environment [1,2]. However, for this to be true, attention must be paid to the activities undertaken during the manufacturing process and after the planned end of the product’s life cycle [3,4,5]. Nowadays, even technologies created as environmentally friendly, such as DSSCs, require the production of some hazardous substances. Traditionally, the production of DSSCs used toxic materials, including metal-based dyes, such as ruthenium and platinum counter electrodes [6,7]. Currently, there are also some trials to produce DSSCs from non-toxic materials, such as natural, plant-based dyes with graphite counter electrodes, but this kind of solution achieves very low efficiencies—up to 1% [8]. Thus, new solutions for increasing the possibility of clean production and effective recycling for DSSCs are required.

These synthetic dyes/contaminants containing azo (−N═N−) segments and benzene or naphthalene compounds make up the majority of all industrial waste contaminants water (>50%). They are the wider problem, not only due to the contamination that comes from the production of solar panels [9]. Synthetic dyes are also used in a variety of food components to increase the appearance and attractiveness of packaged foods without improving their nutrient quality [10]. Further, azo dyes could be degraded to generate numerous amine variants, which can generate a range of medical concerns, particularly in youngsters, such as infections, immunological inhibition, urinary difficulties, and DNA fragmentation. The Allura Red AC (AR) dye (C_18_H_14_N_2_Na_2_O_8_S_2_, molar mass: 496.4) is an artificial food coloring ingredient used in a variety of meat and seafood items, artificial sweeteners, smooth sugary drinks, candies and jellies, dried food, and numerous bread and cow milk goods [11]. According to World Health Organization (WHO) toxicological studies, the AR color may also be dangerous to people and cause serious illness if consumed in large quantities. This AR pigment was already demonstrated to have probable hazardous and malignant consequences, as per the United States Food and Drug Administration (FDA), and is currently prohibited in Switzerland, Belgium, Denmark, and France, as well as being not recommended for young people in the UK. Azo dyes have alkaline earth characteristics, allowing them to investigate anionic features via dissociation of the acidic family and cationic components through the complex formation of non-ionic building blocks or amino groups, which is a concentration function dependent on the presence of hydroxyl, carboxyl, sulfoxyl, or amino gatherings [12].

As a result, scientists have concentrated their efforts on the evacuation of dye from water sources. With the degradation of impurities from water sources, many approaches like the oxidation process, electrocoagulation, liquid–liquid excavation, thermal treatment, catalyst supports, and electrostatic attraction have been examined. Adsorption and photocatalytic degradation have both been shown to be cost-effective and reliable processes that may be applied in a large-scale process. Whenever the crystallite size of the adsorption process is lowered to the nanoscale level, important variations in biochemical, electromechanical, physiological, and optical characteristics have been reported [13,14]. Larger surface area and changed porous arrangement are the most noticeable alterations, both of which boost the substances’ adsorption capabilities. Karunakaran et al. [15] found that a combination of a semiconductor (TiO_2_, ZnO, CuO, and others) with a dielectric material (Al_2_O_3_) resulted in synergistic effects, which boosted photocatalytic efficiency. Organic contaminant photocatalytic degradation was also thoroughly explored.

**Figure 1:**
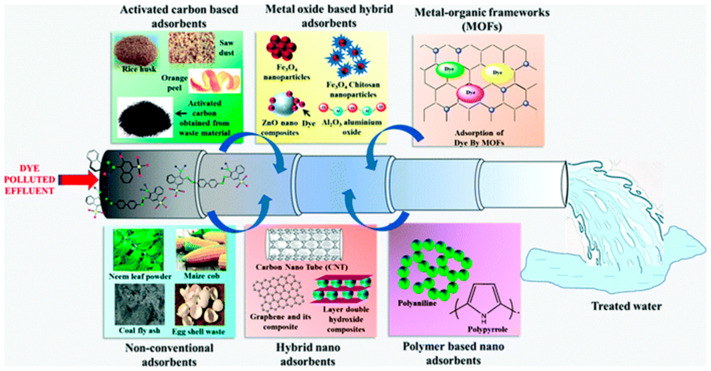
Schematic presentation of water pollution from dye wastewater, the adsorptive removal of dyes using various adsorbents, and the production of treated water (reprinted with permission from [16]).

**Figure 2:**
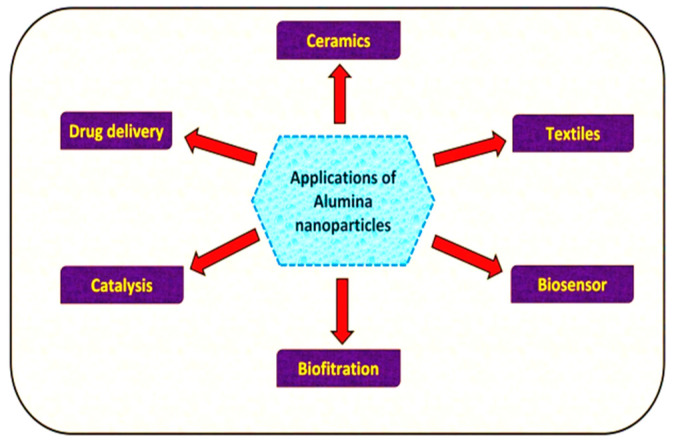
Various applications of Al_2_O_3_ nanoparticles.

A schematic diagram of water pollution from dye wastewater, the production of treated water, and adsorptive removal using various adsorbents is shown in Figure 1. Due to their vast variety of uses in several industries, aluminum oxide (Al_2_O_3_) nanoparticles (NPs) have received a lot of interest. Moreover, ceramic materials relying on Al_2_O_3_ are frequently utilized in the commercial economy due to their distinctive qualities, which include excellent mechanical properties, impact resistance, and exceptional chemical inertness. Al_2_O_3_ has been employed in the hydrocarbon business to make monomeric compounds and remove factory pollutants. At around normal body temperature, Al_2_O_3_ remains a nontoxic and organic solvent for all commonly used chemical compounds; it has high durability and may be honed to a single layer. In the literature, many alloying elements of Al_2_O_3_ have been documented. The critical issue of environmental damage, which includes pollution of the atmosphere, groundwater, and land, poses significant risks to public health and the well-being of all living organisms, particularly humans. In response to these challenges, numerous efforts have been made to develop effective, environmentally friendly, and long-term technologies for removing synthetic dyes and pollutants from freshwater sources. This article reviews the various approaches taken to address the issues in detail, assessing the potential ecological impacts of various technologies. This review intends to contribute to the development of strategies that strike a balance between technological advancements and environmental preservation in the ongoing battle against pollution by shining a light on these initiatives. Due to their remarkable photocatalytic performance, good solubility, and durability, transformation oxides are thought to be a rather intriguing type of catalyst for purifying sewage in a simple, efficient, rapid, as well as environmentally benign manner [16,17,18]. Synthetic colorants are among the most prevalent contributors to groundwater contamination in industries such as manufacturing, fabric, papers, skincare, paintings, and printmaking. Industrial and construction dye ingredients, in fact, comprise various harmful chemical materials and have become a significant determinant of overall water quality (around 20%) alongside pigments, coloring agents, nutrition colorants, and other chemicals [19]. Researchers around the globe are increasingly confronted with problems related to the decontamination of surface water commodities. Fabrics, papers, pigment precursors, leather tanning, pharmacological, and fiber scrubbing sectors, amongst many others, release environmental contaminants like benzidine and naphthalene, as well as other volatile chemicals, into groundwater sources in the environment. The textile sector and its organic effluents are the primary contributors to polluted air across the planet. Metals, salts, lubricants, sulfides, formaldehyde, and other substances are often used in dyestuffs to increase absorption in fibers [20,21]. Highly toxic and hazardous dye-containing harmful byproducts are dumped into the water supply. Various applications of Al_2_O_3_ nanoparticles (NPs) are shown in Figure 2.

The main aim of this article was to conduct a literature review and present solutions for increasing the possibility of clean production and effective recycling of DSSCs, especially the application of Al_2_O_3_-based materials. This work discusses the recent developments in Al_2_O_3_-based materials for the organic degradation or adsorption of dyes as a possible solution to reduce pollution from DSSC technology production. Moreover, Al_2_O_3_ shows excellent activity in organic dye degradation/adsorption since it has a large surface area.

## 2. Photocatalytic Activity of AL_2_O_3_ NMs

In current history, catalyst sewage treatment has received much interest. Nevertheless, there are only a few publications that demonstrate the use of alumina NMs produced from waste material as a nanoscale catalyst for wastewater remediation [22,23]. At 45°C, various aspects of alumina NMs were examined for their catalytic activity to decolorize and degrade fundamental greenish 4 (BG4) dye remedy (100 mg/L) at normal pH with a catalyst dosage of 1.0 g/L with the existence of H_2_O_2_ as an activator in the appearance of H_2_O_2_. In catalyzed reactions, alumina NPs serve as a catalyst area (with multiple diverse classes) for converting H_2_O_2_ to oxidative stress, which then aids dye breakdown via oxygen radical mechanisms [24].

Dye disintegration starts with H_2_O_2_ breakdown on the electrode material of alumina NMs, which produces OH^•^ ions that aid in dye mineralization. OH^•^ ions, on the other hand, respond with large doses of H_2_O_2_ to create hydro-peroxyl radicals (HO_2_^•^) and water. Although HO_2_^•^ is a strong oxidizer within itself, its oxidation rate is considerably lower than that of the OH^•^ radical, which facilitates conservative chemical changes. As a result, HO_2_^•^ does not assist in the process of removing contaminants of dye molecules, which are only mediated by OH^•^ [25]. The following are the chemical properties that should occur throughout the preparation of alumina NMs:

H_2_O_2_ ------Al_2_O_3_----→2OH
Dye + *OH --------------→ Dye Mineralization Products
H_2_O_2_ + *OH -------------→ HO*_2_ + H_2_O
H_2_O_2_ + HO*_2_ -------------→*OH+ H_2_O+ O_2_
HO_2_+ *OH ------------→ H_2_O + O_2_

## 3. Photocatalytic Activity of RhB

**Figure 3:**
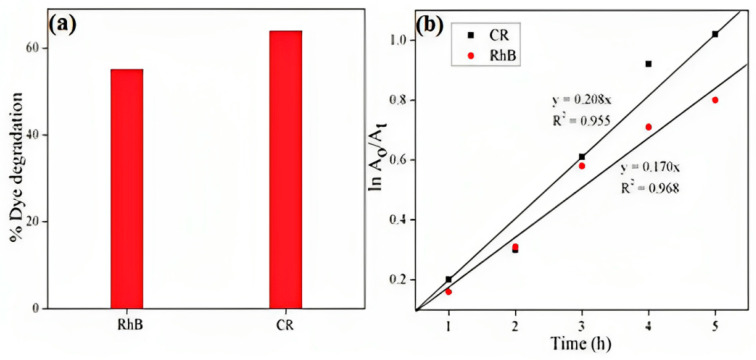
Percentages of dye degradation and pseudo-first-order kinetics, respectively, of the photocatalytic degradation of MG dye (reprinted with permission from [27]).

During sunlight irradiation, photo depletion of RhB and CR utilizing -Al_2_O_3_ NPs was investigated. The reduction in maximum absorbance of -Al_2_O_3_ NPs as a function of irradiation duration for RhB and CR clearly demonstrated how quickly these colors degraded. In the appearance of -Al_2_O_3_ NPs, the color of dye solutions diminished after 6 h of exposure to sunshine [26]. In Figure 3a, the percent disintegration for RhB and CR was determined to be 55.06 percent and 63.9 percent, respectively. RhB (0.170 h^−1^) photo-degradation was lesser than CR (0.208 h^−1^) when exposed to sunshine.

As seen in Figure 3b, the produced Al_2_O_3_ NPs’ photo-electrocatalytic activity was investigated using two functions: adsorbent accompanied by photocatalysts and combined adsorption and photocatalytic degradation underneath sunlight irradiation.

## 4. Adsorption Followed by Photocatalysis

To remove MG dye from the aqueous system, Al_2_O_3_ NPs (in an SH electrolyte, water-ACN solution, with a current of 100 mA, maintained at a high temperature of 1200 °C) were employed. As a consequence of the sunshine immersion time, the typical maximum absorbance of MG at 620 nm was measured. To achieve an adsorption–desorption optimum, the dye solvent Al_2_O_3_ NPs were kept illuminated [28,29]. The resulting mixture was subjected to direct sunlight for additional photo-degradation followed by 1 h of response in blackness. The absorption edge at 620 nm gradually reduced in concentration after expanding the irradiation duration. In the complete lack of a photocatalyst, the dye dispersion was initially irradiated using sun energy before being mixed with the Al_2_O_3_ NP photocatalyst [30]. In dark circumstances, just 12% of the dye was immobilized for 1 h, but photocatalysis during daylight irradiation for 5 h resulted in a 32 percent degradation. The catalyst nanoparticles were heavily coated with organic molecules throughout this adsorption mechanism in the blackness that may have blocked sunlight, leading to less MG breakdown. Once sunlight illuminated these Al_2_O_3_ NPs, electron–hole pairs were formed, interacting with water to generate hydroxyl and superoxide radicals, which broke the bonds within the organic compounds [31].

## 5. Photocatalytic Effect of Implemented Oxides

**Figure 4:**
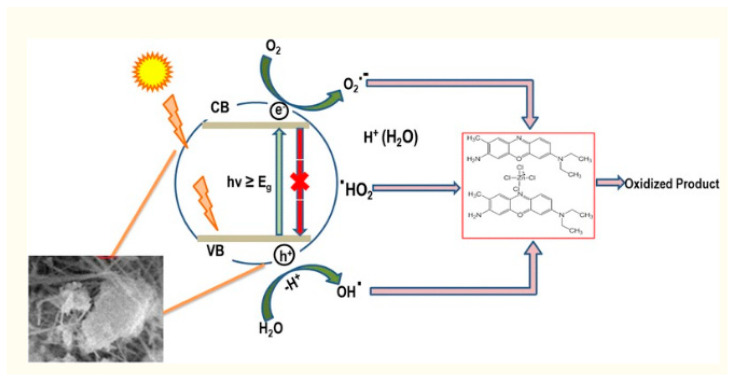
Photographic perception of photo-degradation by Al_2_O_3_-doped Mn_3_O_4_ nanomaterial (reprinted with permission from [35]).

Figure 4 illustrates how the apparent light source from the ultra-band discrepancy leads the valence band electron to be transferred to the conduction band, leaving the electronic conductivity intact [32,33,34].

The electron–hole pair migrates to the NM layer to take part in the electrochemical process. Because the conduction band of the NM is almost iso-energetic and also has a negative potential of oxygen, some of the photo-generated charged particles reassemble with holes in the VB, and some are reassigned to the exterior, where neighboring molecular oxygen can scrounge charged particles from the NM’s conduction band and regenerate them into a superoxide anion revolutionary (O2^•−^). The superoxide reactive next interacts with a proton to produce hydrogen peroxide (H_2_O_2_), which is accompanied by the hydroxyl radical (^•^OH). In addition, the hole (h+) in VB produces hydroxyl radicals (^•^OH) in the watery solution. The deterioration (oxidation) of the compound is caused by repeated assaults of O2^•−^ and ^•^OH agents on pollution components (chemical dyes). Additional hydrogen peroxide could also be added to the reaction process, which has the significant advantage of producing hydroxide ions. The charged particles must be distinguished as much as possible in order for a successful photocatalytic reaction to occur. Furthermore, with the use of photoluminescence, the dye component BCB immobilized on nanocomposites could be promoted to an active state. Following that, photo-excited dye particles can be implanted into the nanoparticle’s conduction band via photo-oxidation. Furthermore, photo-created holes in the valence band of nanostructures destroy dyes (via oxidation) [36].

## 6. Photo-Degradation of Malachite Green

The specimen with (80% Fe, 10% Al, and 10% Mn) had the maximum photo reactivity, with a 98 % breakdown in the visible region; the specimens with (90% Fe, 5% Al, and 5% Mn) and (80% Fe, 15% Al, and 5% Mn) had 97 and 92% degradation, respectively. Following exposure to UV radiation, the specific dye degradation percentages were observed to be 72%, 75%, and 49% for specimens A (80% Fe, 10% Al, and 10 % Mn), B (90% Fe, 5% Al, and 5% Mn), and C (80% Fe, 15% Al, and 5 %Mn), respectively. In the visible region, there was considerable deterioration [37]. The as-synthesized nanocomposite yielded a favorable outcome. Because Fe^3+^ and Mn^3+^ had identical ionic geometries in respective maximum spin mode, that is 0.645, the greater deterioration in the visible region of the spectrum light occurred owing to an unbalanced amount of d electrons in the 3d orbital of Fe and Mn when Fe^3+^ was substituted with Mn^3+^. This result is significant because electronic structure is a critical factor in photocatalytic activity and one of the issues that prevents hematite from becoming an effective electrode material. There were minor variances in the visible range degeneration outcomes of every specimen.

Since Fe_2_O_3_ operates like a server in each of the observations, it accounted for at least 80% of the total, whereas the percentages of Mn_2_O_3_ and Al_2_O_3_ varied somewhat from 5% to 15%. Boosted Mn_2_O_3_ and alumina concentrations between 5% to 10% elevated photocatalytic ability between 97 to 98 % according to deterioration data. If the alumina concentration was increased by 10% to 15%, there was a reduction in deterioration [38]. During ultraviolet light irradiation, specimens A and B revealed comparable deterioration, with sample A showing a small improvement in disintegration. This decomposition outcome of sample C, which includes the lowest proportion of alumina of all the as-produced samples, showed a substantial variation. The deterioration rate underneath UV radiation climbed as the alumina concentration increased from 5% to 15%.

The efficiency of a photocatalytic activity to degrade in water solutions is determined by its capacity to consume light and the efficiency of electron sequence interactions. This capacity encourages the development of oxidizing hydroxide (OH^•^) molecules, which need a band gap value that is appropriate in comparison with the intensity of incoming photons. The amount of charge carrier interaction is required to increase the formation of hydroxyl ions. The disadvantage of TiO_2_ is that it possesses a significant charge separation ratio, as the live duration of energy levels is in the range of 10^−9^ to 10^−12^ s, preventing the synthesis of oxidizing compounds. Another disadvantage is that it has a 3.2 eV band gap, which is around UV rays (300 nm). This restricts its employment in daylight with a large percentage over 380 nm since its capacity to gather photons in the optical range is reduced [39,40].

The proficient degradation of dyes by using a photocatalyst in the aqueous medium depends upon its ability to absorb light and its lower rate of recombination of electron–hole pairs. This ability promotes the generation of oxidizing hydroxyl (OH^•^) radicals that require suitable band gap energy compared with that of incident photons. Enhancing the generation of hydroxyl radicals is dependent on the charge carrier recombination rate. The drawback of TiO_2_ is that it has a high recombination rate, as the lifetime of excited states is of the order of 10^−9^ to 10^−12^ s, which, in turn, suppresses the formation of oxidizing species. The other drawback is that it has a wide band gap of 3.2 eV, which lies at near-UV radiation (300 nm). This reduces its ability to absorb the photons in the visible spectrum, meaning that Ephoton ≤ 380 nm has limited application in sunlight due to having a major portion of band gap energy above 380 nm. The matter of recombination inhibition and light harvesting in the visible region can be made effective through the modification of the surface of TiO_2_ via composite formation. The charge isolation would be initiated by the interlayer charge exchange amongst the permissible energy levels [41]. Collaboration charge transition across the intermediate energy bands of the baseline element, TiO_2_, and the materials of the produced compounds, CuO, Al_2_O_3_, and ZrO_2_, is one of the proposed methods of mutation prevention.

## 7. Green Synthesis of Al_2_O_3_ Nanoparticles

Many techniques have been widely employed to generate Al_2_O_3_ NPs in recent years; nevertheless, they have a few drawbacks such as the greater expense of these procedures and the fact that they are not environmentally friendly because they use hazardous chemicals and poisonous limiting agents. Green chemistry techniques for the synthesis of Al_2_O_3_ NPs have been used to overcome these limitations and boost the accuracy of such techniques. These strategies are recyclable, less energy-intensive, and eco-friendly [42]. While synthetic stabilizers are used more often than natural ingredients, these compounds are hazardous to the environment and individual welfare. Biomolecules such as amino acids, enzymes, proteins, steroids, phenols, tannins, sucrose, and flavonoids, which are generally available in pharmacological herbal extractions and are environmentally friendly, are required for the stability of Al_2_O_3_ NPs. Physiological methods are utilized to generate Al_2_O_3_ NPs in diverse morphological features and dimensions from plant components including leaves, seed fruit, and flowers. The aqueous solubility heterocyclic components are primarily responsible for nanoscale production and stability. Following that, the biosynthesized NPs must be described utilizing a variety of approaches [43].

## 8. Photo-Degradation of Dyes

**Figure 5:**
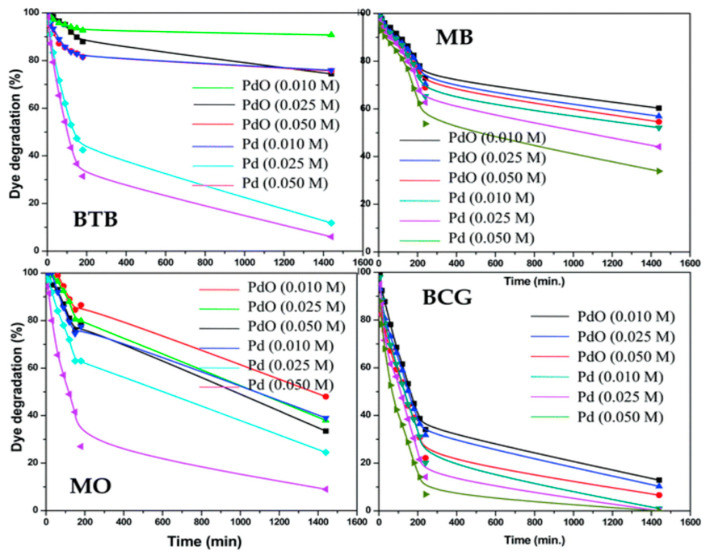
Percentages of organic dye photo-degradation by Pd-γ-Al_2_O_3_ and PdO–Al_2_O_3_ catalysts (reprinted with permission from [47]).

**Figure 6:**
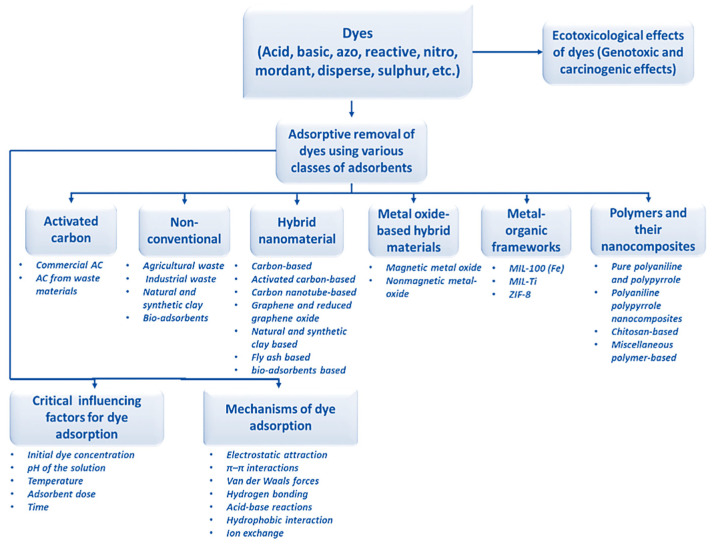
Flow chart showing the classes of dye molecules, their ecotoxic effects, and the removal processes using different classes of adsorbents together with their sub-categories (reprinted with permission from [48]).

Using Rhodamine B, methylene blue, and methyl orange as experimental dyes, the photocatalytic activities of the produced Al_2_O_3_-TiO_2_ and ZrO_2_-TiO_2_ nanostructured materials under UVA light were examined. Methyl orange is an azo dye, methylene blue is a heterocyclic dye, and rhodamine B is a xanthene dye. The percentage of organic dye photo-degradation by Pd-γ-Al_2_O_3_ and PdO–Al_2_O_3_ catalysts is shown in Figure 5. These catalysts have diverse chemical formations and correspond to three separate groups. Figure 6 shows the decay patterns of the three dyes by the two nanomaterials under UV-A light [44,45,46].

The photocatalytic breakdown of methyl orange dye by the two produced nanomaterials is obviously slow, as seen by the decomposition characteristics. However, in comparable investigational settings, the remaining two colors fade gradually. Adsorption of the dye on the irradiated areas of the nanocomposites might be one cause. The dye component must be deposited on the irradiated area of the nanocomposites for efficient translocation of photo-induced holes and reactionary oxygen agents from the semiconductor to the compound.

It is important to note that medium accumulation on an exposed interface differs from that on an unlit layer. That is, daylight and darker adsorption are not the same. The adsorption of methyl orange on the illuminated nanocomposites is probably scarce, but the adsorption of rhodamine B and methylene blue on the exposed nanocomposites is significant. While the photographic production of electron combinations in the produced nanocomposites requires UV-A light, daylight equally causes dye photocatalytic degradation. As a consequence, the detected rhodamine B photo-degradation involves dye-sensitized energy partition in the photocatalytic mechanism, which results in the degradation of dyes.

## 9. Photocatalytic Test

During synthetic UV illumination, the photocatalytic activities of the produced substances for phenol photo-degradation were investigated. As a control, the TiO_2_-P25 Degussa substance was used. The photolysis and obscurity responses were also carried out as already stated. Further investigation was carried out to confirm that the phenol was never dragged out of the mixture by the air filtration. During the photo-oxidation, gloominess, and preservation trials, it was obvious that the phenol content stayed stable [49]. This research demonstrated that the primary way for phenol to be transformed is for daylight to react to photocatalysts. The photocatalytic properties of Al_2_O_3_: Ce^3+^ /Ce^4+^ materials showed that pure Al_2_O_3_ and AC5.0 exhibited similar optical properties. This discovery demonstrated that applying 5.0 wt. % Ce^3+^/Ce^4+^ to the Al_2_O_3_ photocatalyst did not boost its photocatalytic efficiency. When 1.0 wt. % Ce^3+^/Ce^4+^ was placed over Al_2_O_3_, though, phenol precipitation attained 94 percent after 3 h of processing. Amongst the catalysts described previously, both of the two other substances produced phenol mineralization. In comparison with the adjusted Al_2_O_3_:Ce^3+^/Ce^4+^ samples, the TiO_2_-P25 corresponding sample had the least phenol oxidation. Considering that the AC1.0 sample had the greatest phenol mineralization, we investigated its photo-degradation activity with additional chemicals like 4-chlorophenol, p-cresol, and 4-nitrophenol in mixtures of around 40 ppm to determine if the derivative reactive group in the para region affected the activity efficiency [50]. In using a 40 ppm dilution mixture and 200 mg of the maximum effective photocatalyst, the concentration of the absorption peaks in the UV area associated with the electronic interactions of the aromatic ring diminished with the duration of ultraviolet light treatment, indicating that the photo-oxidation activity of organic compounds occurred.

It is evident that without the catalysts, none of the four organic compounds showed significant photo-oxidation. Based on our findings, the photo-degradation of the components occurs in the following sequence: 4-nitrophenol < phenol <pcresol < 4-chlorophenol; this discovery might be interpreted by the capacitive impact of the electro-attractor or provider of the side groups at the para location [51]. The chloride segment is an excellent acceptor; therefore, it is an excellent electro-influencer; the nitro compound is also a useful releasing group, but because of the operational component, this cluster offers molecular durability, making ring formation much tougher and the speed of the process sluggish. The generation of precursors in the reaction system causes the dislocation of the absorbance band in the 4-chlorophenol compound. The UV–Vis analysis of concentrations of certain auxiliaries, including hydroquinone, catechol, and benzoquinone, was performed to illustrate the probable mixtures formed throughout the photo-degradation of phenol; based on this article, these auxiliaries are the components that might be created [52]. However, during the photocatalytic activity of phenol decomposition, a spectrum in the range of 282–301 nm emerges, a spectrum focused at 287 nm emerges with the 4-chlorophenol molecule, and a spectrum in the region of 290–310 nm occurs with the p-cresol compound. These bands emerge to equate to the creation of precursors like p-benzoquinone, catechol, and hydroquinone, which are formed in modest amounts as a combination. In the reference column, there is a UV–Vis graph of p-benzoquinone, catechol, and hydroquinone. The photo-degradation activities of the compounds 4-nitrophenol, phenol, p-cresol, and 4-chlorophenol were evaluated using the standard substance P25 (Degussa). Because the tempo of the response is crucial in the early reaction periods, the percentage of photo transformation was evaluated after 90 min of response. The AC1.0 substance was found to have higher activation than P25, indicating that these elements are novel in the field and are also more efficient than the main prevalent standard semiconductor P25.

## 10. Recycling Methods

Recycling dyes used in DSSCs is a critical pursuit in the quest for sustainable energy technologies. DSSCs represent a promising avenue in renewable energy, harnessing the power of sunlight through photoactive dyes to generate electrical energy. However, the environmental impact of the dyes employed in these solar cells has prompted the exploration of recycling methods as a means to mitigate potential harm [53].

Dyes in DSSCs play a pivotal role in the conversion of solar energy into electricity. Commonly, these dyes consist of complex organic molecules or heavy metals such as ruthenium. While these components are essential for the efficient functioning of DSSCs, they pose environmental challenges when the cells reach the end of their operational life. Disposal of these solar cells without proper recycling measures can result in the release of harmful substances into the environment, contributing to pollution of soil and water [3,54].

The prospect of recycling dyes used in DSSCs holds significant scientific and environmental implications. The recycling process involves the extraction and recovery of dyes from used solar cells, with the aim of reintegrating these materials into the production of new DSSCs. Scientifically, this endeavor requires a thorough understanding of the chemical composition of the dyes, the fabrication processes of DSSCs, and the development of effective methods for dye extraction and purification [55].

One of the primary challenges in recycling DSSC dyes lies in the diverse range of materials used in these solar cells. The dyes can vary in their chemical structures, and some may contain intricate molecular arrangements that demand sophisticated recycling techniques. Researchers are engaged in exploring methods that ensure a high yield of recovered dye while preserving the structural and functional integrity necessary for solar cell applications. This entails developing processes that can efficiently separate the dye from other components, such as electrodes and electrolytes, without compromising the quality of the recovered material [56].

Efforts to recycle DSSC dyes align with broader initiatives to create a circular economy for solar technologies. By recycling and reusing materials, the environmental footprint of solar cell production can be reduced, contributing to a more sustainable energy ecosystem. The importance of such initiatives is underscored by the growing global demand for clean and renewable energy sources [6].

Moreover, the recycling of DSSC dyes holds economic benefits. As the demand for solar energy continues to rise, the recycling of essential components becomes not only an environmental responsibility but also a strategic approach to resource management. Recovering valuable materials from used solar cells can reduce the reliance on virgin resources, making solar energy technologies more economically viable in the long run [7].

While the possibility of recycling dyes used in DSSCs is promising, it necessitates a multidisciplinary approach. Collaboration between materials scientists, chemists, engineers, and environmental experts is essential to address the technical intricacies and environmental considerations associated with the recycling process. Additionally, regulatory frameworks and industry standards need to be established to ensure the safe and responsible recycling of DSSC components. Figure 5 shows a flow chart of dye molecules, their ecotoxic effects, and the removal processes using various classes of adsorbents together with their critical influencing factors, sub-categories, and probable adsorption mechanisms.

## 11. Applications

### 11.1. Organic Pollutant Elimination

Extremely harmful and dangerous organic substances (e.g., chemical dyes) build in the atmosphere as a result of fast community accumulation and intensive industry. Some of the most intriguing strategies for pollution rehabilitation are photo-electrocatalytic breakdown or breakdown of organic contaminants. Typically, wide-bandgap semiconductors (e.g., TiO_2_ and ZnO) used as traditional photocatalysts for the removal of chemical impurities are mainly photoactive under ultraviolet rays that contribute to just 4% of the overall renewable energy. The combination of wide-bandgap diode photocatalysts with Al nanoparticles has been displayed to be a viable strategy for extending the light absorption spectrum between UV and illumination. For instance, an Al/TiO_2_ photocatalyst compound has been created that has better photocatalytic activity over uncovered TiO_2_ when it comes to the removal of methylene blue (MB) using visible light irradiation. 

The mean optical degradation ratio for Al/TiO_2_ combinations, on the other hand, is 8.18 × 10^−4^ s^−1^, which is nearly 150 times higher than that of the naked TiO_2_ layer. The following is the reason for the increased photo activity of the Al/TiO_2_ nanocomposite: The LSPR was drastically shifted to the observable area of the band in this work by placing gratings of Al nanorod dimers on the interface of TiO_2_ surfaces. As a result, during interface plasmonic non-radiative recreation, warm electrons on Al nanorod dimers may be stimulated by the visible region and transferred into the surrounding TiO_2_ layer, promoting the rapid production of covalent bonds in TiO_2_. The Al nanorod dimers provide holes for MB compounds, while the TiO_2_ layer deposits electrons into immersed oxygen, causing MB to degrade into lesser particles [57].

### 11.2. Other Applications

Other applications of the organic dye degradation and adsorption techniques reviewed in this article include water splitting and wound healing. Photo electrical and chemical hydration breaking to create H2, which generates sustainable and pure energy from daylight, is seen to be a realistic option for fulfilling upcoming energy demands. In this field, hematite (-Fe_2_O_3_) has received a lot of interest as a possible photographic electroactive. Its optical sensor has a potential optimum solar to hydrogen (STH) reliability of 15.5 percent, although the STH performance obtained in practice is far lower than the potential limitation. A plasmonic Al nanoscale may be created coupled with hematite electrodes to considerably boost optical absorbance and minimize electron couple generation to optimize STH yield and reach remarkable water-separating quality [58]. Moreover, Si-Al-Fe_2_O_3_ core–multishell (CMS) nanowire (NW) photoelectrodes use Al as a new component of plasmonic photoelectrodes to improve water splitting effectiveness. Si-Al-Fe_2_O_3_ CMS NWs have much higher absorbance in optimum circumstances than Si-Fe_2_O_3_ core–shell (CS) NWs owing to Al plasmon resonance. Furthermore, Al is important for localizing the collected photoelectron fluctuation inside the hematite shell, ensuring that the charges are created to approach the activation region with little recirculation. The improved absorption and decreased rearrangement lead considerably to an STH productivity of 14.5 percent, which is almost 93 percent of the rated capacity for bulk hematite [59].

Throughout the medication processes, wound healing operation for Al_2_O_3_ NPs was demonstrated in medicated mice using an excision injury framework comparable to a regulation method. 

With increasing inflammatory response, oxidative exposure was a key factor in retarded tissue repair and created substantial levels of ROS. Inflammatory response, vasculature, tissue migrations, preliminary framework production, granulation tissue formation, and re-epithelization are only a few of the physiological and microbiological processes associated with inflammatory treatment. The healing mechanism necessitates a complex interplay between inflammatory cell types, pharmacological intermediaries, embedding structural components, and micro-environmental cellular functions, all of which are influenced by a variety of mitogens and cytotoxic activity elements. Conversely, recovery deficiency is characterized by prolonged cellular penetration and granulation morphogenesis, diminished angiography, lowered extracellular matrix, and organization, all of which were observed in mice that were not given NPs. The cause for this change is assumed to be the formation of elevated quantities of responsive oxygen agents and elevated rates of apoptosis, which disrupt the metabolic activity of keratinocyte endothelial cell types, fibroblasts, and collagen [60]. Iron oxide nanoparticles are recognized for their small size, large surface area, and considerable interactions with carbon nanotubes, enabling them to easily penetrate the hair shaft, leading to improved and restructured hair regeneration in mice treated with NPs.

### 11.3. Environmental Impact

The environmental impact of the dyes used in these cells requires a thorough investigation that considers all stages of the dye life cycle. The extraction and synthesis of ingredients used in dye manufacturing cause the first phases of environmental effects. Many colors used in DSSCs are generated from organic compounds or metal complexes, which require time-consuming extraction methods. The extraction of organic compounds and metal complexes has the potential to disturb habitats and create ecological imbalances. For example, the extraction of certain metal components, such as ruthenium or other rare metals commonly utilized in DSSCs, may include mining activities that lead to deforestation, soil degradation, and ecological damage. Furthermore, the manufacturing of these dyes entails chemical processes that use a lot of energy and produce waste. 

To reduce the carbon footprint of dye manufacturing, energy sources must be carefully considered. Transitioning to renewable energy for these processes might assist in reducing the environmental impact of dye production. The toxicity of the dyes’ constituents is a critical feature of their environmental impact. Certain dyes may include materials or chemicals that, if released into the environment, can endanger ecosystems. Heavy metals employed in some dye formulations, for example, can be persistent pollutants, potentially accumulating in soils and water bodies and harming flora and animals. To guarantee that dyes do not cause long-term environmental impacts, their toxicity must be assessed.

Aside from issues of toxicity, the extraction of raw materials for color synthesis may contribute to resource depletion. Demand for commodities such as metals and organic molecules can place a strain on limited resources, threatening world biodiversity. Sustainable sourcing practices, such as the research of alternative materials with lower environmental impact, are critical for minimizing natural resource depletion. Beyond color synthesis, the manufacturing process itself contributes to the overall environmental impact. If non-renewable energy sources are used, the energy-intensive aspect of DSSC production contributes to greenhouse gas emissions. In order to match DSSC technology with broader sustainability goals, renewable energy must be integrated into the production process.

**Figure 7:**
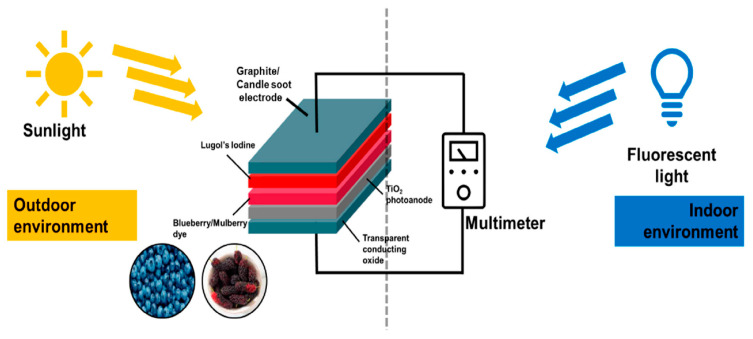
Schematic diagram of the natural dye-based DSSCs measured in indoor and outdoor environments. The diagram was produced as an engineering teaching kit (reprinted with permission from [62]).

In Figure 7, a schematic diagram of the natural dye-based DSSCs measured in indoor and outdoor environments is shown. The diagram was produced as an engineering teaching kit. Another aspect of the environmental impact of DSSC dyes is waste manufacturing. Byproducts and waste materials generated during the manufacturing process must be properly managed. To reduce the environmental impact of DSSC manufacturing, proper waste disposal and recycling practices must be implemented [61]. Furthermore, dye durability and stability are essential aspects throughout the operational life of DSSCs. Dyes that degrade quickly or emit toxic byproducts during their lifetime may have a greater environmental impact. Ongoing research focuses on creating dyes with higher stability and lower environmental impact. Consideration of the entire life cycle of DSSCs, including deployment and eventual disposal, is critical for a thorough assessment of environmental effects. The end-of-life period presents material disposal and associated environmental issues.

## 12. Conclusions

This review presented the present solutions for increasing the possibility of clean production and effective recycling of DSSCs, especially with the use of Al_2_O_3_. In the environmental decontamination of organic dyes, metal oxides play an important role. The main source of water pollution, which harms water bodies, living beings, and ecological systems, is contamination with heavy metals and dyes. Al_2_O_3_ showed high removal percentages for dye removal in the literature reviewed, with most of these adsorbents showing removal percentages of nearly eighty percent, which is why it can be considered an excellent adsorbent alternative for adsorption. Adsorption is a fast and best method to remove organic and inorganic pollution. This study may also offer a theoretical basis to find new, highly efficient photocatalytic processes for organic dyes using the full explanation of the analysis and identification of degradation products of organic dyes. The application of an Al_2_O_3_-based catalyst photocatalytic method to multi-component organic dyes requires additional investigation since organic dyes in real-world wastewater are in the form of a composite. Moreover, the majority of existing photocatalytic research has avoided the toxicity issue of the degradation of intermediates and instead mainly focused on the rate and effectiveness of the degradation of targeted organic dyes. It is indeed difficult to conduct a study on the topic since the factors that produce a reliable level of synthesis, which are necessary in order to use photocatalysis more effectively, are unknown. An accomplishment in this area might be a major step forward for pollutant removal.

## Data Availability

Not applicable.
